# Chemo-immunotherapy of colon cancer with focused ultrasound and *Salmonella*-laden temperature sensitive liposomes (thermobots)

**DOI:** 10.1038/s41598-018-30106-4

**Published:** 2018-08-30

**Authors:** Kalyani Ektate, Maria Cristina Munteanu, Harshini Ashar, Jerry Malayer, Ashish Ranjan

**Affiliations:** Center for Veterinary Health Sciences, Oklahoma State University, Stillwater, Oklahoma USA

## Abstract

Using attenuated *Salmonella* that efficiently homes in solid tumors, here we developed thermobots that actively transported membrane attached low-temperature sensitive liposome (LTSL) inside colon cancer cells for triggered doxorubicin release and simultaneous polarized macrophages to M1 phenotype with high intensity focused ultrasound (HIFU) heating (40–42 °C). Biocompatibility studies showed that the synthesized thermobots were highly efficient in LTSL loading without impacting its viability. Thermobots demonstrated efficient intracellular trafficking, high nuclear localization of doxorubicin, and induced pro-inflammatory cytokine expression in colon cancer cells *in vitro*. Combination of thermobots and HIFU heating (~30 min) in murine colon tumors significantly enhanced polarization of macrophages to M1 phenotype and therapeutic efficacy *in vivo* compared to control. Our data suggest that the thermobots and focused ultrasound treatments have the potential to improve colon cancer therapy.

## Introduction

**Figure 1:**
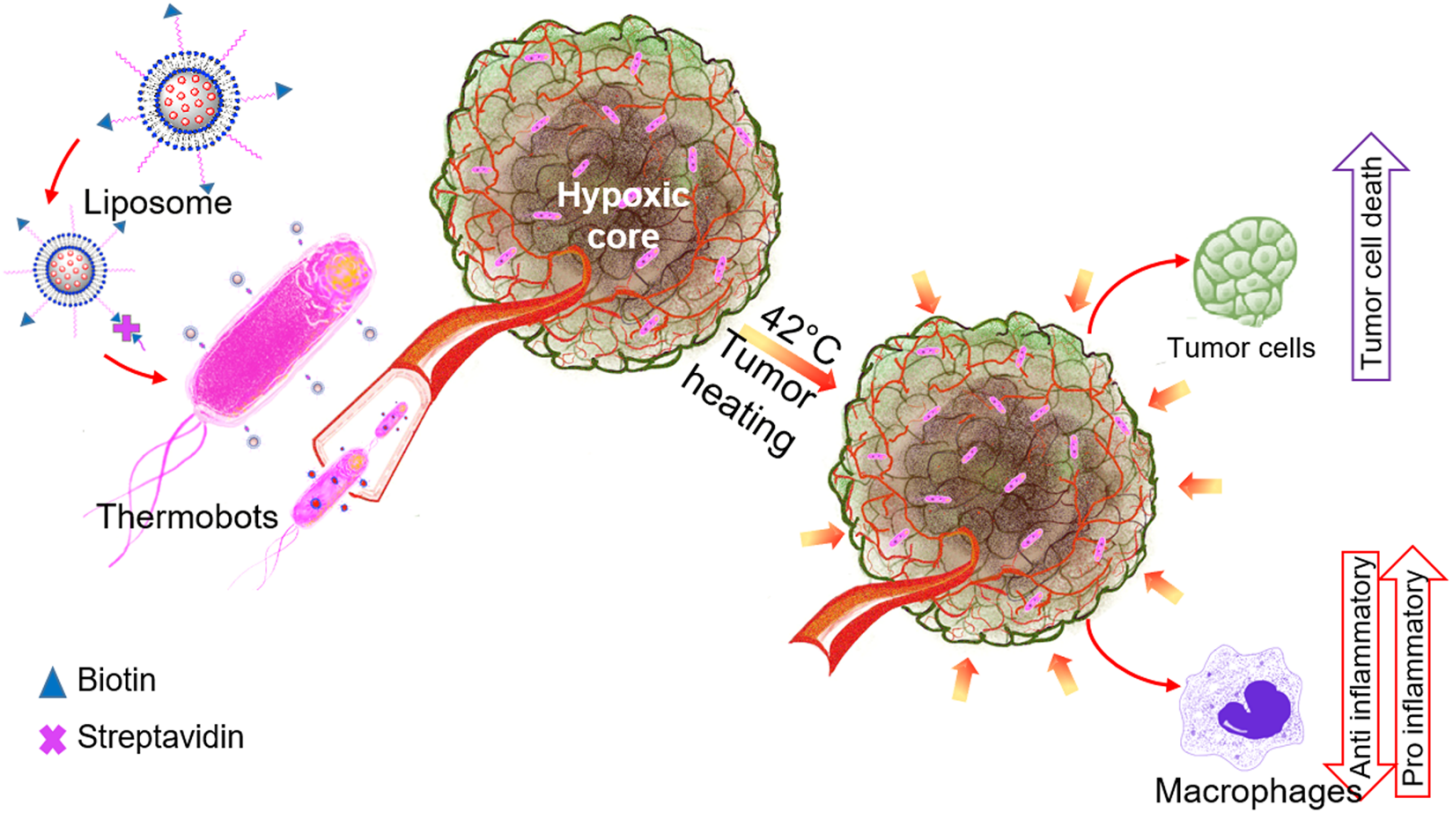
Schematic of thermobot and heat-combined chemo-immunotherapy approach against colon cancer.

Colorectal cancer (CRC) is the second leading cause of cancer-related deaths with an incidence rate of 40% in the United States and an average 5-year survival rate of < 15% in metastatic disease^1^. Chemotherapy of metastatic CRC has modest efficacy and is typically associated with toxic side effects^2^. Recent clinical trials suggest that patients with refractive CRC tumors can benefit from the enhancement of the tumor-immune system interactions^3–5^. Specifically, tumor-associated macrophages (TAMs) that release mediators to help shape the adaptive immune response in CRC are described to be a key link to effective immune control and escape of cancerous cells^6,7^. TAMs can adopt an M1 (classically activated) and M2 (alternatively activated pro-tumor) phenotypes. Classically activated M1 macrophages produce proinflammatory cytokine-like IL1-beta, and TNF-alpha to enhance the efficacy of chemotherapeutics and induce tumor regression. In contrast, alternatively activated macrophages (M2) secrete protumoral and chemoresistance inducing cytokines like IL-10, and TGF-beta^8,9^. Although the role of macrophages in CRC has been extensively investigated, therapeutic strategies that modulate the M1/M2 macrophage phenotypes in real-time to improve chemotherapy outcomes is yet to be developed successfully. Our central hypothesis is that the combination of thermosensitive liposome-laden *Salmonella* (Thermobots; TBs) and tumor heating (~40–42 °C) with high intensity focused ultrasound (HIFU) can induce macrophage-related immunological changes to synergistically enhance colon chemotherapy (Fig. 1).

Attenuated bacterial microorganisms such as *Salmonella*, *Clostridium*, *Listeria*, *Shigella* and *Escherichia coli* are known to self-propel and home within a solid tumor^10–16^. In particular, *Salmonella typhimuriu*m (YS1646) shows high chemotaxis towards the serine, ribose, and aspartate that are produced by the quiescent and hypoxic cells within the benign and metastatic tumors^11,17,18^. Salmonella infects cancerous cells via the expression of a type-III secretion system (TTSS)^19^, and utilizes the membrane lipopolysaccharide (LPS) to induce secretion of proinflammatory cytokines, nitric oxide and eicosanoids^20^. Loading Salmonella with low-temperature sensitive liposomes (LTSL) can provide additional benefits^21–25^. LTSLs contains the lyso-pc lipid that undergoes a structural and chemical phase change to achieve triggered doxorubicin (Dox) delivery in tumors in response to HIFU heating^22,24,26–30^. Trafficking LTSLs using Salmonella will improve tumor targeting, localization and the maximum tolerated Dox dose without inducing severe systemic toxicity. Furthermore, membrane LPS, a classical activator of M1 macrophages can help overcome the chemoresistance and immunosuppressive tumor microenvironment to directly improve LTSL therapy. Thus, we posit that the chemo-immune effects in the tumor microenvironment with LPS and Dox has the potential to significantly enhance malignant CRC therapy^31^. Towards this goal, our objectives in this study were to optimize TB design principles, understand chemo-immunomodulatory mechanisms plus minus HIFU heating, and characterize therapeutic efficacy in a mice model of colon cancer. Our specific interest in choosing HIFU in the TB combinatorial regimen stems from its unprecedented tumor-targeting capabilities, and ability to induce mechanical and thermal stress that is key to the removal of tumor immunosuppression^32–35^. Thus, characterizing the effects of TBs with HIFU should give important insights on the impact of pro- and anti-inflammatory CRC tumor microenvironment on chemotherpay effects in tumors.

## Results

### Salmonella efficiently loads and maintains the therapeutic efficacy of LTSLs

**Figure 2:**
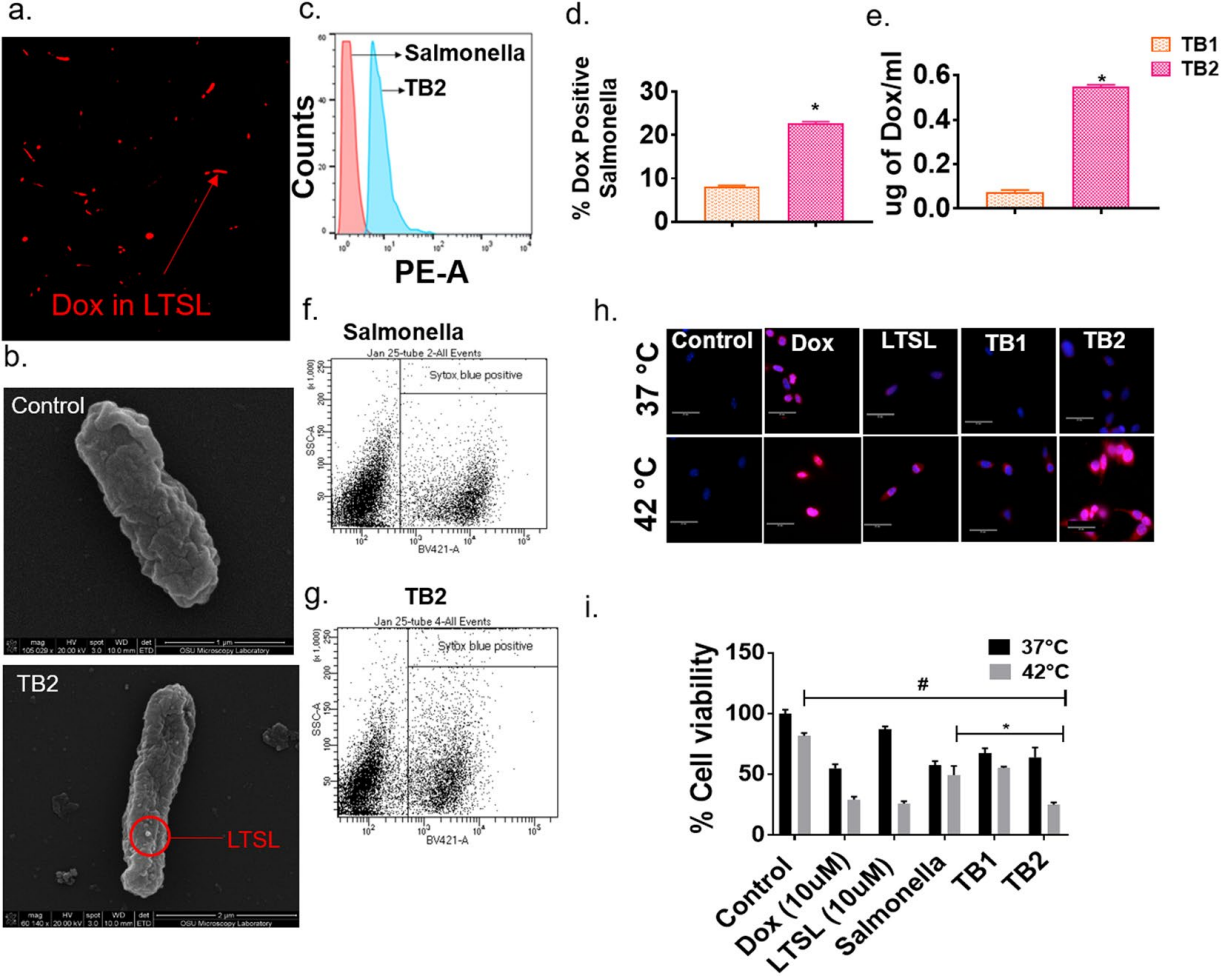
*In vitro* characterization of thermobots. (**a**) Epifluorescence microscopy showed LTSL presence on the Salmonella membrane as indicated by red fluorescence of Dox. Salmonella alone (not shown) was used as control and didn’t demonstrate any fluorescence. All images were captured with the 60x oil immersion objective lens; (**b**) SEM image of Salmonella showed punctate liposomal dots on the membrane; (**c**) Histogram plots indicated a gradual increase in the MFI for bacterial population positive for dox (depicted in blue) compared to the control (depicted as red peak); (**d**,**e**) Dox quantification by spectroscopy and flow cytometry demonstrated 2–4 fold greater drug loading with streptavidin-biotin crosslinking for TB2 compared to TB1 (p < 0.0001); (**f**,**g**) Viability of TB showed no major shift in the dead cell population compared to controls in the FACS density plot with SYTOX staining; (**h**) Streptavidin-biotin cross linking improved intracellular dox delivery for TB2 compared to passively loaded TB1.Confocal images were captured at 40x magnification. Dox is shown in red and nuclei in blue; (**i**) Cellular viability of C26 colon cells post treatment with dox, LTSL and TBs enhanced bacterial killing at 42 °C compared to 37 °C; Values represent mean ± SE, and were normalized to control samples at 37 °C. (n = 6; ANOVA followed with tukeys).

Prior research has shown that stealth liposomes passively adsorb on the bacteria membrane by electrostatic or Van der Waals type attraction^36^. However, the detachment of the liposomes from the bacterial membrane in a circulatory environment can be a concern. To address this, we synthesized TBs by attaching LTSL onto Salmonella membrane using Biotin-Streptavidin chemistry^18,37^. Epifluorescence microscopy showed the presence of fluorescent LTSL-Dox (red) overlapped with the rod-shaped *Salmonella* membranes using the cross-linked methodology (Fig. 2a). The presence of liposomes were additionally confirmed by SEM imaging where LTSL was found to be present as punctate dots on the bacterial membrane (Fig. 2b). In general, an average of 15–20 liposomal dots (n = 25) attached on to the *Salmonella* membrane. In contrast, *Salmonella* alone appeared smooth. Next, we quantified the Dox loading potential of TBs by flow cytometry and fluorescence spectroscopy. Flow cytometry indicated a gradual shift in the mean fluorescence intensity of TBs in the Dox filter range relative to Salmonella alone suggesting effective membrane attachment (Fig. 2c). Notably, compared to TBs that were passively incubated with LTSLs (TB1; MFI: 8.16 ± 0.014), the peak shift was significantly higher by ~2.5 fold for Biotin-Streptavidin attached TBs (TB2; MFI: 21 ± 0.14; Fig. 2d; see Table 1 for lipid compositions). The fold increase in fluorescence intensity matched with Dox quantification by spectroscopy. A ~7.5 times more Dox/bacteria for TB2 (540 ± 80 ng/ml) compared to TB1 (70 ± 10 ng/ml) was noted (Fig. 2e). Most importantly, the enhanced loading of Dox didn’t induce significant decrease in bacterial viability compared to *Salmonella*, and a 70–75% viability was noted for both the TB and untreated control bacteria (Fig. 2f,g).

**Table 1:** Cross-linking schemes for Thermobot (TB).

Thermobot (TB)	Lipid Composition	Diameter ± SD (nm) (LTSL)	Poly dispersity Index ± SD (LTSL)	Zeta Potential ± SD (mv) (LTSL)	Biotin-Streptavidin reaction
TB1	DPPC, MSPC, and DSPE-mPEG2000	170.41 ± 3.1	0.125 ± 0.02	−27.41 ± 4.3	No
TB2	DPPC, MSPC, DSPE-mPEG2000, and DSPE-PEG2000-Biotin	177.67 ± 3.3	0.104 ± 0.01	−34.34 ± 13.3	Yes

We also characterized the intracellular trafficking and cytotoxicity of TBs. C26 colon cancer cells were incubated with Dox, LTSL, TB1, and TB2 in the presence and absence of heating (~42 °C). Confocal microscopy showed significantly enhanced nuclear localization of Dox in the heated cells. Specifically, upon 4 h post infection, the uptake and nuclear localization of Dox from TBs were significantly higher at 42 °C compared to body temperature (Fig. 2h). The increased TB2 uptake and Dox release with heat correlated with enhanced therapeutic efficacy (~80% C26 killing) compared to TB alone (~50%). As expected, the efficacy of TB1 and TB2 was relatively higher compared to control at body temperature (~35–40% killing), however, TB2 demonstrated higher potency with heat (~80% killing) compared to TB1 (60%), presumably due to enhanced Dox transport inside the colon cell (Fig. 2i). Based on these data, TB2 was selected for further *in vitro* and *in vivo* evaluation.

### TB and heat treatment enhances pro-inflammatory gene expression *in vitro*

**Figure 3:**
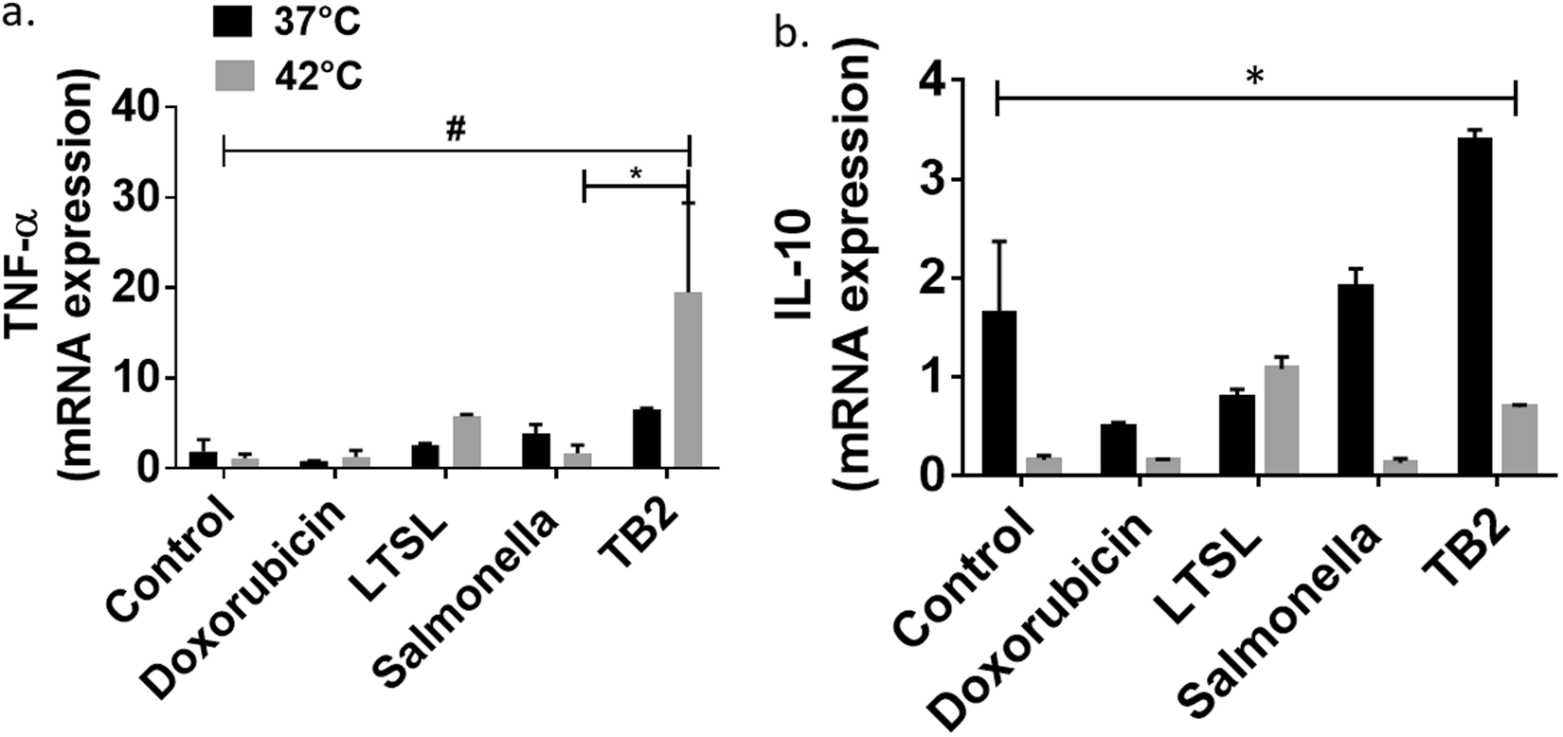
Impact of TB/heat treated C26 condition media on RAW264.5 macrophage cytokine gene by qRT-PCR. (**a**) TB plus 42 °C achieved significantly higher TNF-α gene expression compared to controls, dox and LTSL treatment at 37 °C and 42 °C; (**b**) Significant drop in IL 10 expression at 42 °C compared to body temperature was noted for all treatments. Values represent means ± SE (n = 3) for each treatment, ^#,*^p < 0.05.

LPS endotoxin present in the outer leaflet of *Salmonella* interacts with immune cells to promote the secretion of proinflammatory cytokines^20^. Whether the LPS immune effects synergizes with TB/heat therapy is not known. To investigate this, the conditioned media from C26 cells that were treated with Dox, LTSL, Salmonella, and TB2 in the presence and absence of heat was added to RAW264.7 macrophages, and the pro-inflammatory (TNF-α) and anti-inflammatory (IL10) cytokine gene expression was assessed (see Table 2). Heat plus TB treatment significantly enhanced TNF-α in the macrophages compared to all treatment groups at 37 °C (Fig. 3a). This increase in pro-inflammatory cytokine was also accompanied by a decrease in IL10 expression in the heated groups compared to controls (Fig. 3b). These results suggest that adding heat to TB or Salmonella treatment increases pro-inflammatory properties of colon cancer cells.

**Table 2:** qRT-PCR primers sequences.

qPCR primers	Species	Sequences
mGapdh-forward	*Mus musculus*	CATCACTGCCACCCAGAAGACTG
mGapdh-reverse	*Mus musculus*	ATGCCAGTGAGCTTCCCGTTCAG
mTnf- alpha-forward	*Mus musculus*	CACCACCATCAAGGACTCAA
mTnf-alpha-reverse	*Mus musculus*	AGGCAACCTGACCACTCTCC
mIl10-forward	*Mus musculus*	CGGGAAGACAATAACTGCACCC
mIl10-reverse	*Mus musculus*	CGGTTAGCAGTATGTTGTCCAGC

### TBs and HIFU therapy enhances therapeutic efficacy *in vivo*

**Figure 4:**
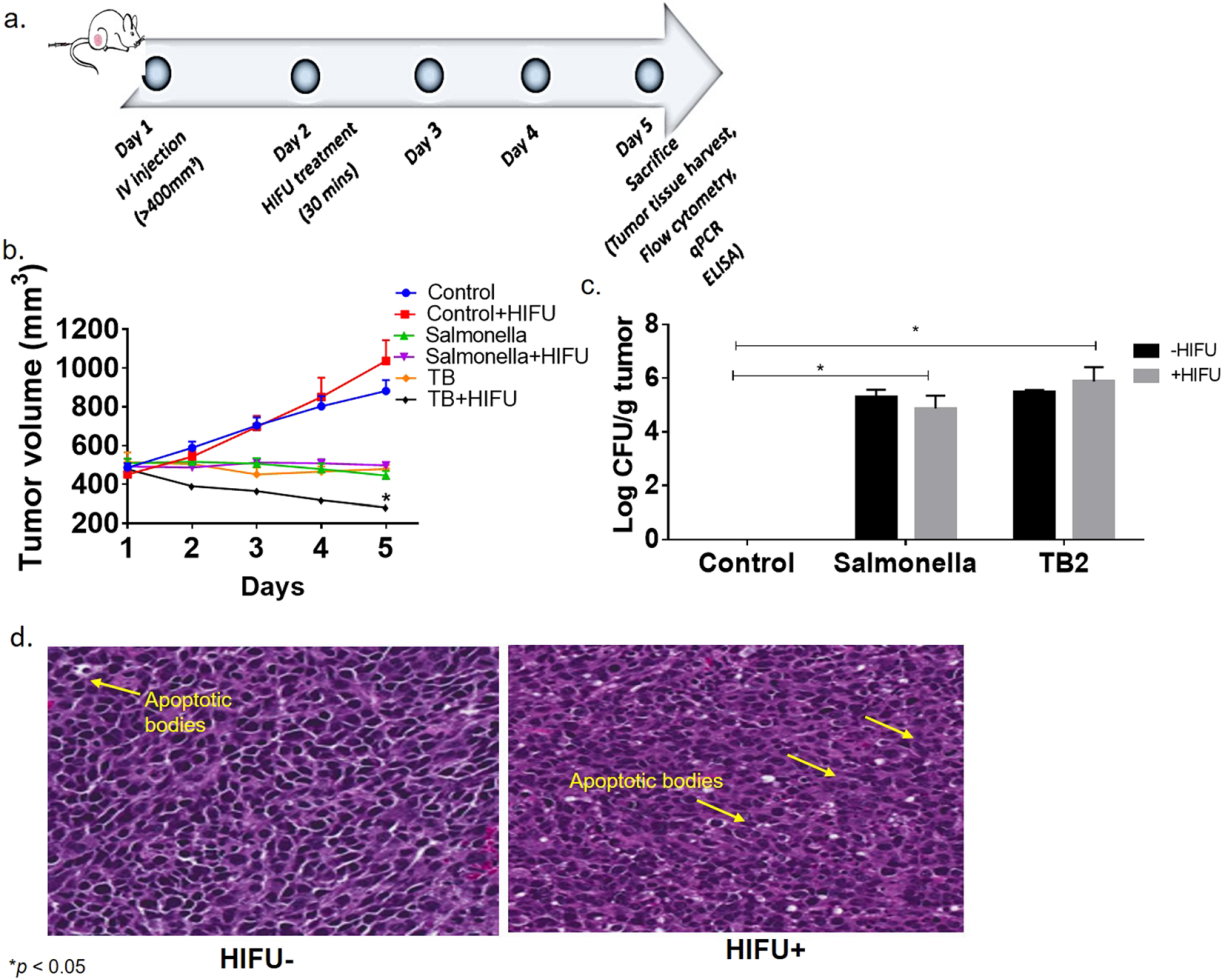
*In vivo* efficacy in C26 colon tumor model following HIFU/TB treatment. (**a**) Female Balb/c mice (n = 6/group) were inoculated with 0.5 million C26 cells subcutaneously in the flank region. Mice were treated at a volume of >400 mm^3^ mice with a single dose of saline/Salmonella/TB (IV). 24 h post bacterial injection, HIFU treatment was administered for 30 min. Treated mice were sacrificed on day 5 post HIFU and the organs were processed for immunopathology; (**b**) Significant tumor regression with TB/HIFU compared to all other groups was noted over 5days. (**c**) Colony forming unit (CFU) per gram of tumor enumerated by plating tumor homogenates on LB agar suggested efficient tumor colonization with TB in the presence and absence of HIFU. (**d**) H & E section of mice tumors suggested an increase in the apoptotic bodies for HIFUed tumors in comparison to untreated tumors. Values represent means ± SE for each treatment; *p < 0.05 from control (ANOVA followed by Tukey’s).

The efficacy of TB/HIFU was evaluated by tumor growth and histological measurements. Briefly, when the C26 tumors reached >400 mm^3^ volume, the mice were treated with TBs and Salmonella (Fig. 4a). Enumeration of tumor colonization rates for Salmonella and TBs suggested that the drug loading doesn’t impact tumor infiltration (Fig. 4c). 24 h post injection, a single non-cytotoxic HIFU treatment (~42°) for ~30 min was administered, and tumor volumes were measured for 5days post treatment. A pronounced increase in tumor volume (400–500 mm^3^) was observed for HIFU similar to untreated control mice (Fig. 4b). *Salmonella*-alone treatments induced significant tumor volume reduction, however, the effects were not stimulated by the HIFU treatment. In contrast, the addition of HIFU heating to TB treatment resulted in greater suppression of tumor growth rates compared to Salmonella or TB alone, likely due to triggered Dox release in colon cells. Additional characterization of the HIFU treated tumors by H&E staining suggested a slight increase in the apoptotic bodies compared to controls, indicative of tumor damage (Fig. 4d).

### TB and HIFU treatment enhances M1 phenotype, and infiltration of CD4 T-helper cells

**Figure 5:**
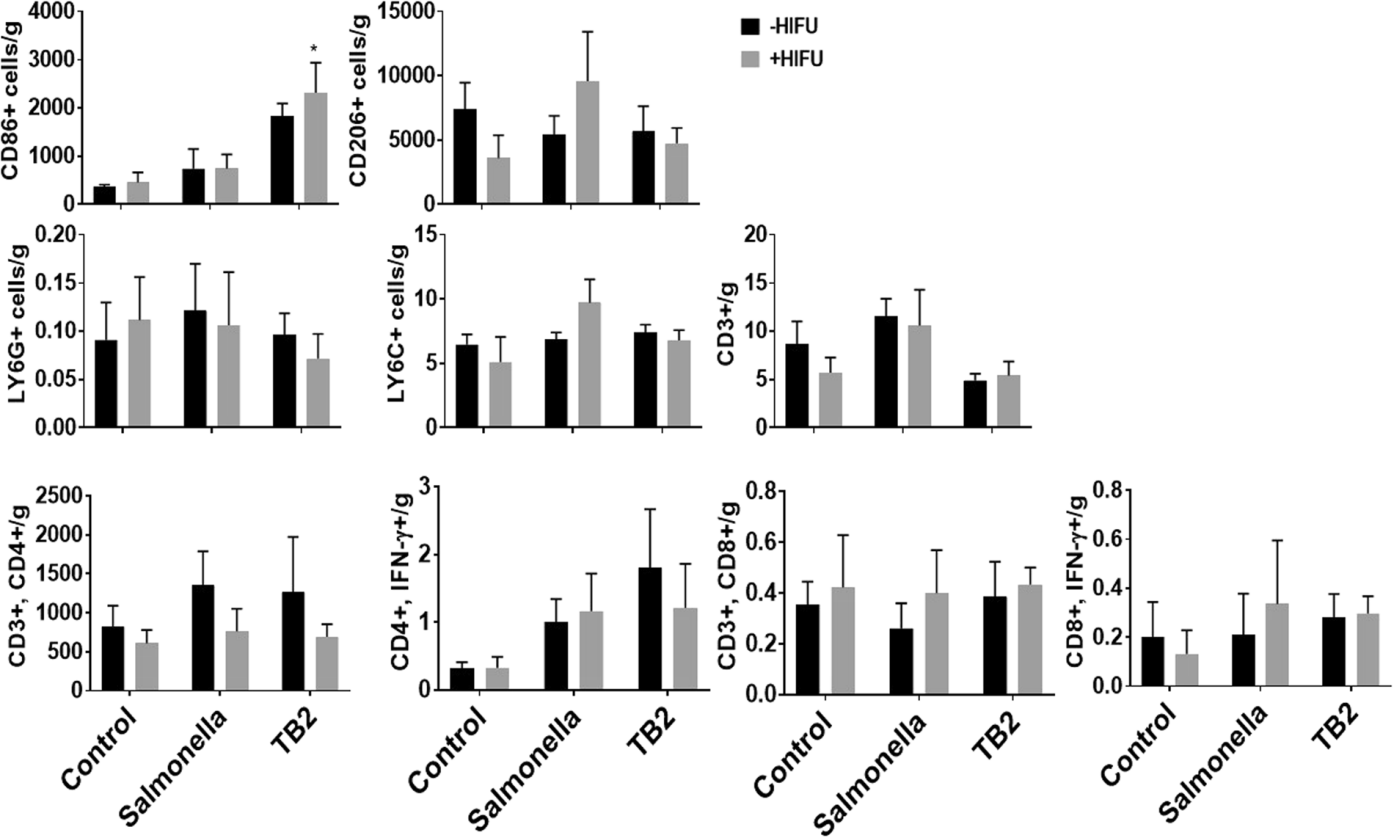
Evaluation of infiltration of immune cells in colon tumors by flow cytometry. Tumor cells were labelled *ex-vivo* with following panels: macrophages (CD11b+, CD86+/CD206+), MDSC’s (CD11b+, Ly6C+/Ly6G+) and T cells (CD3+, CD4+/CD8+, IFN-γ+). Percentage of M1 infiltrating macrophages was significantly increased in colon tumors following TB and HIFU therapy compared to other groups. Percentage of CD3+ CD4+ IFN-γ T-cell was increased for TB and Salmonella relative to control group, however, the population of CD8+ cells were unaltered. The influx of granulocytic (Ly6G+) or monocytic (Ly6C+) myeloid-derived suppressor cells (MDSCs) was not altered by single TB/HIFU treatment. Data (n = 6, means ± SEM) represents percent positive population of immune cells in the tumors (*p < 0.001).

The mechanisms underlying the *in vivo* immunomodulatory effects of TB/HIFU combination was analyzed in the harvested tumor by flow cytometry. TB/HIFU combination caused the highest increase in the expression of M1 macrophages when expressed as per gram of tumor compared to all other treatments. HIFU heating also enhanced the M1 phenotype in Salmonella alone group, however, this was accompanied with a proportional increase in the M2 phenotype. In contrast, TB/HIFU M1 induction didn’t result in a compensatory increase in M2 phenotype, thereby skewing the M1/M2 population towards a higher M1 phenotype (Fig. 5). We also characterized the activation of the effector adaptive immune cells (e.g., interferon (IFN)-*γ*-producing CD4^+^ T cells for Th1 cell type I antitumor response, and cytotoxic CD8^+^ T lymphocytes (CTL) mediated cell lysis)^38,39^. IFN-γ activates expression of MHCII and co-stimulatory molecules in the antigen presenting cells, and promote the Th1 differentiation of CD4+ T cells to increase immunogenicity of tumor cells. An IFN-γ driven Th1 type response is generally accompanied by autocrine activation of macrophage via IL-12 signaling^40,41^. Compared to untreated control, both Salmonella and TB infection of colon cells achieved a ~2–3 fold increase in the helper (CD3+, CD4+) cells and interferon gamma (IFN-γ) expressing CD4+/CD8+ cells/g tumor with and without HIFU, suggesting the possible role of this pathway in macrophage polarization dynamics (Fig. 5). In contrast, the population of cytotoxic/killer (CD3+, CD8+) T cells was not altered by the treatments, presumably due to an early sacrifice of mice (day 5), and a delay in activation of memory cell population post first treatment. The immune microenvironment was also characterized for changes in the influx of either granulocytic (Ly6G+) or monocytic (Ly6C+) myeloid-derived suppressor cells (MDSCs). Monocytic (mMDSC) and granulocytic (gMDSC) subsets facilitate tumor disseminations by inducing T-cell suppression via nitrosylation of T-cell receptor, MHC-II complex, reactive oxygen species production, and differentiation into M2-macrophages^42^. The MDSCs didn’t show significant changes at the time of tumor harvest between various treatment groups, indicating that this may not be predominant mechanisms of tumor control and dissemination in our model system.

### Serum cytokine levels detected by ELISA

**Figure 6:**
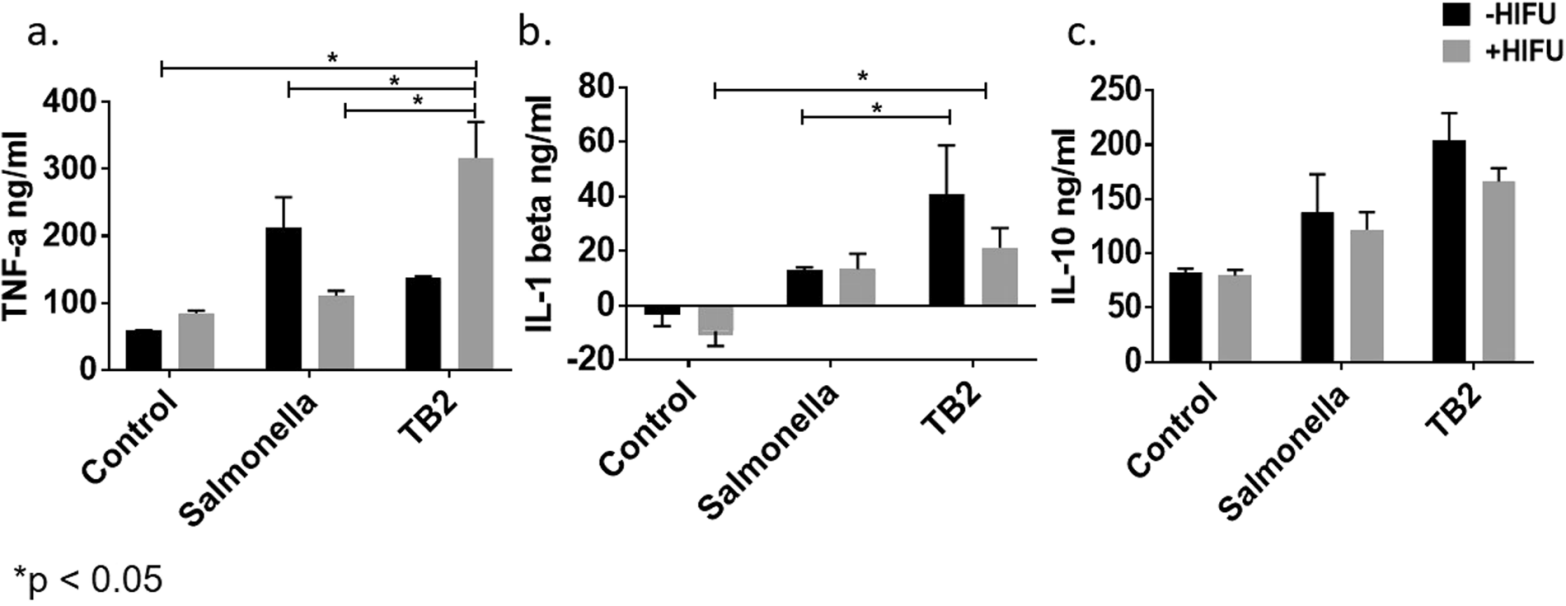
Enhancement of pro-inflammatory cytokines in serum with TB treatment. ELISA was performed to detect the serum cytokine levels of TNF-α, IL1-β and IL-10. (**a**) TB2 + HIFU increased TNF-α serum cytokine level compared to control, *Salmonella* and TB treatment at 37 and 42 °C; (**b**) IL-1β serum levels increased by 2–3 fold for TB treatment compared to controls; (**c**) IL-10 levels were increased by 2–3-fold by HIFU/TB compared to controls. Values represent means ± SEM (n = 4, *p < 0.05).

ELISA was performed to detect the serum cytokine levels of TNF-α, IL1-β and IL-10 following TB/HIFU, Salmonella and HIFU treatments. TNFα, and IL-1β are elevated in CRC, and thus are potential targets of antagonists in phase I/II clinical trials^43^. Significantly enhanced TNF-α serum cytokine level (316 ± 53 ng/ml) was noted for HIFU plus TB mice tumor groups compared to untreated control (58.3 ± 1.15 ng/ml), HIFU (84.7 ± 3.93 ng/ml) and *Salmonella* (110.48 ± 7.82 ng/ml) treated mice (Fig. 6a). TB and Salmonella treatments also increased IL-1β serum concentration by ~2–3 fold compared to untreated controls (Fig. 6b). Additionally, a 1.5–2-fold concurrent increase in IL-10 cytokines with TB/HIFU (180.5455 ± 16.64 ng/ml) and Salmonella/HIFU *Salmonella* ± HIFU (121.84 ± 16.10) compared to HIFU (79.7 ± 5.09) alone was also noted (Fig. 6c).

## Discussion

In this study, the feasibility of combining bacteriolytic chemotherapy with TB in combination with HIFU heating was assessed. We hypothesized that TB will induce tumoricidal effects, while also aiding in the antitumoral immunomodulation with HIFU heating. To enhance *in vivo* stability, TBs were created by attaching LTSLs onto *Salmonella* membrane actively using Biotin-Streptavidin chemistry^18,37^. Biotin-Streptavidin is amongst the strongest known non-covalent protein-ligand reaction (Ka = 2.5 × 1013M-1)^44,45^. This method of cross-linking resulted in a ~7.5 fold greater membrane binding of LTSL compared to passive incubation without impacting bacterial viability or cellular uptake (Fig. 2c–i). Also, in contrast to some prior reports where *Salmonella* achieved drug deposition mostly in the perinuclear region, TBs upon combination with heat achieved efficient drug delivery in both the cytoplasm and the nucleus of C26 cells^46^. Most likely, the heat treatment of cells enhanced membrane fluidity and protein rearrangement in the cells, aiding the Dox transport kinetics molecules^47^. An additional unexplored but key finding was the initiation of pro-inflammatory phenotype in macrophages following exposure to TB/Heat treated 10% conditioned media. 10% condition medium was chosen *in vitro* to mimic *in-vivo* condition of colon tumors where the peritumoral macrophages are likely exposed to such level of tumor-derived cytokines^48^. A high expression of TNF-α, and decreased expression of IL10 was noted in the macrophages. As macrophages and dendritic cells are the main producers of TNF-α and their activation typically shift the macrophage phenotype from pro-tumoral M2 to M1^48,49^, we assessed M1 and M2 macrophage population in the treated tumors by flow cytometry. Both TB and TB/HIFU treatment enhanced M1 phenotype compared to all other treatment groups (Fig. 5), thereby suggesting that this is a predominant mechanism of tumor regression *in vivo* compared to controls.

The generation of pro-inflammatory tumor environment by TB and HIFU stimulated the adaptive immune response to cause a moderate increase in the population of T-cells in the tumor (Fig. 5). A low-to moderate increase in T-cell infiltration can be partly due to the utilization of late stage poorly vascularized and necrosed murine colon tumors (>400 mm^3^). Despite this, an overall increase in IFN-γ-secreting CD4 cells in tumors was noted for Salmonella and TB groups (Fig. 5). CD4+ IFN-γ cells are important for T helper (Th1) immunity and prevents autoimmunity. The proliferation of Th1 type cells following TB/Salmonella likely points toward a protective response against the bacterial infection in our murine model. The increase in CD4 cells were not accompanied by a concurrent increase in the recruitment of CD8 cells. The expansion and recruitment of memory cells to the tumor typically requires >7 days, and the tumor bearing mice were sacrificed by day 5 post treatment, and this may explain our findings.

Finally, the TB and HIFU treatment didn’t enhance the population of MDSCs that stimulate the tumorigenic processes in a tumor (Fig. 5). MDSCs can produce IL-10, and TNF-α to influence their cross-talk with macrophage and tumor cells^50,51^. Our data points towards a significant increase in the IL-10 serum protein level, and this may be MDSC mediated since *in vitro* studies suggested a decrease in IL-10 in the absence of MDSCs. (Figs 3 and 6). Although a detailed interplay of cytokines and immune-cell cross-talk was not fully characterized, we believe that the pro-inflammatory TNFα mediated macrophage activation likely mitigated the IL10 immune suppressive effects of MDSCs to some extent *in vivo*. Studies to delineate the role of IL10 on MDSC tumor modulatory pathways especially in the context of Doxorubicin, HIFU and Salmonella combinatorial therapy are currently in works.

## Conclusion

We report for the first time novel thermobots for chemoimmunotherapy with HIFU heating. TBs enriches M1 macrophage phenotype and Th1 population and achieve triggered release in tumors to enhance therapeutic effects. This combinatorial technology has the potential for treatment of CRC.

## Materials and Methods

### Materials

The lipids monostearoyl-2-hydroxy-sn-glycero-3-phosphocholine (MSPC), 1,2-dipalmitoyl sn-glycero-3-phosphocholine (DPPC), and 1,2-distearoyl-sn-glycero-3-phosphoethanolamine-N-[methoxy (Polyethylene glycol)2000] (DSPE-mPEG2000) were obtained from Corden Pharma Corporation (Boulder, CO, USA). DSPE-PEG (2000)-Biotin was purchased from NANOCS (NY, USA), and Streptavidin (97062–810) was bought from VWR (PA, USA). EZ-link NHS-LC-Biotin and trypsin-EDTA, MatTek 35 mm glass bottom dish with 10 mm glass diameter (NC0445924), SYTOX™ Blue dead cell stain (S348557), TRIzol reagent (15596018), and DNase I (EN0525) was obtained from Thermo Scientific (MA, USA). iScript™ Reverse Transcription Supermix for RT-qPCR (1708840) was purchased from Bio-Rad (CA, USA). Doxorubicin (Dox) was acquired from LC Laboratory (MA, USA). The PD-10 column was obtained from GE Healthcare Life Sciences, (Buckinghamshire, United Kingdom). Ethylenediaminetetraacetic acid (EDTA) and 4-(2-Hydroxyethyl) piperazine-1-ethane sulfonic acid, and N-(2-Hydroxyethyl) piperazine-N′-(2-ethanesulfonic acid) (HEPES) were acquired from Sigma (St. Louis, MO). Luria broth (LB) agar and broth were purchased from BD (NJ, USA). Fetal bovine serum was procured from Atlanta Biologicals (GA, USA), and Penicillin/Streptomycin was acquired from Invitrogen (CA, USA). Fluorochrome-conjugated anti-mouse monoclonal antibodies were purchased from BioLegend (CA, USA: APC-CD11b (101212), PE-CD86 (105008), PE-Ly6C (128008), PE-CD4 (100512), APC Cy7-IFNg (505850), Pacific blue-F4/80 (123124), Alexa fluor 700-CD206 (141734), and BD biosciences: FITC-Ly6G (1A8) (551460). UltraComp eBeads were purchased from Fisher Scientific (Hampton, NH). SYBR green reagent (qPCR Master Mix Plus for SYBR green) was purchased from Eurogentec (Liège, Belgium). ELISA kits for TNF-α(MTA00B), IL1-β (MLB00 C) and IL-10 M1000B were purchased from R&D (MN,USA). C26 murine colon cancer cells were kindly provided by the National Cancer Institute, and RAW 264.7 cells were obtained from ATCC. *Salmonella typhimurium* (YS1646) was purchased from ATCC (Manassas, VA, USA).

### LTSL synthesis and characterization

LTSLs (lipid composition: DPPC, MSPC, and DSPE-mPEG2000; molar ratio of 85.3:9.7:5.0) were prepared by hydration of lipid film followed by the extrusion^52^. For cross-linking to *Salmonella* membrane, DSPE-PEG2000-biotin lipid was incorporated in the LTSL membrane (lipid composition: DPPC, MSPC, DSPE-mPEG2000, and DSPE-PEG2000-Biotin; molar ratio of 85.3:9.7:4.0:1). Briefly, lipids were dissolved in chloroform, and the solvent was evaporated. The resulting lipid film was hydrated in citrate buffer (pH 4.0) at 55 °C for 30 minutes and extruded five times through double stacked 200 nm polycarbonate filters. Encapsulation of Dox into the liposomes was carried out using a pH gradient loading protocol as described by Mayer *et al*.^53^. LTSLs were characterized for size (z-average), polydispersity index and zeta potential using dynamic light scattering (DLS) with a 90 plus PALS Nanobrook device (Brookhaven Instruments, Holtsville, NY, USA). Briefly, 10–20 µl of LTSLs were added to 2 ml of PBS in a cuvette, and DLS measurements were recorded at room temperature. An average of five measurements was taken, and the mean size and standard deviation were calculated for the LTSLs. For recording the zeta potential, 10 µl of the liposomes were suspended in 1500 µl of double distilled water, and an average of 5 measurements was taken to record the mean zeta potential.

### Synthesis of LTSL attached *Salmonella* (Thermobot or TB)

A library of LTSL attached *Salmonella* with active and passive cross-linking schemes as given in Table 1 were synthesized^54^. For cross-linking, 3 × 10^8^ CFU of *Salmonella* was suspended in 0.5 ml PBS with 100 μg of EZ link NHS-Biotin (dissolved in 5 μl DMSO) for 45 min at room temperature under mild shaking. Free EZ link NHS-Biotin was removed by centrifuging at 3000 × g for 10 min and washing 2 times with PBS. Cross-linking with LTSL’s was achieved by incubating 3 × 10^8^ CFU of Salmonella with 100 μg of Streptavidin solubilized in deionized water and 100 μl of 22 mM Dox loaded LTSL for 1 h at room temperature. As additional controls, Salmonella was passively co-incubated with 100 μl of 22 mM Dox loaded LTSL for 1 h. Unattached liposomes were separated out by centrifuging the bacterial suspension at 3000 × g for 10 minutes. The bacterial pellet was washed 2x with PBS and resuspended in 500 μl of PBS for further analysis.

### Quantification of Dox in TBs with flow cytometry and spectroscopy

TBs (n = 3; 3 × 10^8^ CFU) were examined in a FACS Aria flow sorter (BD Biosciences, Franklin Lakes, NJ, USA) at an excitation wavelength of 488 nm and a 590/30-nm emission filter using BD FACS Diva 8.0.1 software. Data were computed and compared from the dot *plots* and histogram plot by counting 10,000 single cell events.

For Dox quantification by fluorescence spectroscopy, TBs (3 × 10^8^ CFU) suspended in 0.5 ml of PBS was heated in a water bath maintained at 45 °C for 30 minutes. The bacteria were centrifuged at 3000 × g for 10 min., the supernatant containing the released Dox was collected and the fluorescence was measured at a wavelength of 480/590 nm with Spectramax (CA, USA) microplate reader. Concentrations of the Dox in supernatants were determined from the linear calibration curve using least squares regression method based on the nominal concentration.

### Dox imaging in TB by fluorescence and SEM imaging

Based on flow and Dox loading, TB2 was selected from 5.4. 200 ul of diluted TB was added to cover the glass surface of MatTek dish (10 mm glass diameter) for imaging with 60x oil immersion objective. All imaging was acquired using Olympus ZDC2 IX81 fluorescence microscope equipped with a color CCD camera, cooled monochrome CCD camera, motorized scanning stage, and Metamorph mosaic stitching software. Dox fluorescence was collected and measured at a wavelength of 480/590 nm using a 20 ms exposure time with a custom made a filter (excitation 480/40 nm, emission 600/60 nm, and dichroic 505lp) at 60x APO.

For SEM, TB2 and control *Salmonella* was fixed for 2 h in 2.0% glutaraldehyde in 0.2 M cacodylate at room temperature, rinsed 3x with PBS, and then fixed for 1 hour in 1% OsO4 in 0.2 M (in water). Fixed samples were then washed 3x with PBS and 20 μl of fixed TB3 and *Salmonella* was placed onto a poly-L-lysine coated 12 mm glass slide and incubated for 30 minutes. The slides were washed with ultrapure deionized water. Samples were dehydrated with increasing content of ethanol for 15 minutes. at each concentration (30%, 50%, 70%,90%,95% and 100%). A critical point dryer (BAL-TEC CPD030), was used to remove ethanol from the dehydrated samples. Samples were immediately mounted on stubs with carbon tape and a wafer coated with gold/palladium prior to imaging (Cressington Carbon Coater; Balzers Union MED 010 Au/Pt coater). High-resolution images were captured with FEI Quanta 600 field emission gun ESEM with Evex EDS and HKL EBSD at an accelerating voltage of 20 kV, and a working distance of < 10 mm and the number of nanoparticles attached on the surface of 25 bacteria was counted manually to determine the average number of nanoparticles attached on bacteria.

### Assessment of TB viability by Flow cytometry

5 × 10^7^ CFU of *Salmonella* and eTB’s was diluted in PBS to achieve a final volume of 1 ml for viability analysis with SYTOX blue dead cell stain. 1 μl of SYTOX blue was added to each sample to achieve a final dye concentration of 1 μM followed by 5 min incubation in dark at room temperature^55^. *Salmonella* with no treatment and no SYTOX blue was used as a control for setting up the flow voltage and gate parameters. Samples were analyzed without washing or fixing using a 440/40 nm bandpass filter with BD FACS Diva 8.0.1 software. Data were computed and compared from the dot *plots* and histogram plot by counting 10,000 single cell events.

### Assessment of cellular uptake of TB

2.5 × 10^4^ C26 cells were seeded in glass bottom Petri dishes overnight and were incubated at a multiplicity of infection of 50 with *Salmonella*, TB1 and TB2 at 37 °C or 42 °C for 1, 4 and 12 h. LTSL (containing 0.02 µg of Dox) incubated under similar conditions as TB served as a control. Prior to imaging, cells were rinsed with PBS 2 × , fixed by adding 1 ml of 4% paraformaldehyde to the plates for 15–20 minutes at room temperature. The nucleus was counterstained with DAPI (3uM) for 10 minutes at room temperature and rinsed 2x with PBS. Imaging was performed using Olympus IX81 confocal microscope with the Doxorubicin filter (ex/em of 480/590) and the DAPI filter (ex/em of 365/440) at 40x by randomly selecting multiple regions in the petri dish.

### Evaluation of TB cytotoxicity

Cytotoxicity was assessed for TB at 50 MOI (multiplicity of infection) at 37 and 42 °C. C26 cells (1 × 10^5^ cells/well) cultured in RPMI 1640 media supplemented with 10% Fetal Bovine Serum (FBS) and 1% Penicillin/Streptomycin at 5% CO_2_ and 37 °C were seeded into 96 well flat bottom plate for 24 h. Cells were washed 2x with PBS to remove antibiotic. TBs and *Salmonella* (50 MOI) suspended in no antibiotic and serum-free medium containing 10 µM of Dox was added to the culture well and incubated for 4 h at 37 °C and 42 °C. Extracellular TB were removed by treating with RPMI containing 50 μg/ml of gentamicin for 1 h at 37 °C. Next, the culture media was discarded, well were washed with PBS and re-suspended in 100 µL of cell culture media, and incubated at 37 °C for ~20 h. An *in-vitro* homogeneous, colorimetric method for determining the number of viable cells using the MTT (3-(4, 5-dimethylthiazol-2-yl)-2,5-diphenyltetrazolium bromide) was used to determine any cytotoxic effects of the released Dox. Briefly, 10 μl of 12 mM MTT was pipetted into each well, and the plates were incubated for 4 hours at 37 °C in a humidified 5% CO_2_ atmosphere. The absorbance at 540 nm with Spectramax (CA, USA) microplate reader.

### Immune analysis *in vitro*

#### C26 cell conditioned medium collection

5 × 10^5^ C26 cells /well were treated with 50 MOI of *Salmonella*, TBs and LTSL (containing 50 ng Dox) for 4 h at 37 or 42 °C in RPMI medium (no antibiotic, no serum). Extracellular bacteria were removed by treating with gentamicin (50 µg/ml) for 1 h, and cells were cultured for 6 h in serum free media. Next, tumor cell conditioned media was collected, centrifuged at 1000 rpm for 5 minutes to remove cell debris and the supernatant was stored at −80 °C.

#### Treatment of RAW 264.7 macrophages with colon cancer conditioned medium

0.5 × 10^6^ RAW 264.7 cells suspended in 2 mL media were seeded in 24 well culture plate overnight. Confluent cells were treated for 24 h with 10% C26 conditioned media at 37 °C in 5% CO2 incubator. For RNA extraction, media was discarded and cells were collected in 1 ml TRIzol reagent and stored at −80 °C until RNA extraction was performed.

#### RNA isolation and DNase treatment from RAW 246.7 cells and tumors for gene expression analysis

Total RNA from the RAW 264.7 cells was extracted using TRIZOL according to the manufacturer’s instructions. RNA concentration was measured using NanoDrop ND-100 and 5 µg of total RNA was treated with DNase I according to manufacturer’s protocol. Post DNase I treatment, the RNA was purified with the phenol-chloroform methodology.

#### Reverse Transcription Polymerase Chain Reaction (RT-PCR)

cDNA synthesis was performed using 1 µg DNase I treated RNA per reaction using iScript Reverse Transcription Supermix for RT-qPCR. Real-Time RT-PCR reaction was performed with cDNA diluted 5×.

#### Quantitative Real-Time Reverse Transcription Polymerase Chain Reaction (qRT-PCR)

Relative gene expression for TNFα (M1 cytokines), and IL10 (M2 specific cytokines) was evaluated by qRT-PCR using SYBR green reagent using Applied Biosystem 7500 fast Real-Time PCR instrument and indicated specific primers sequences (Table 2). qRT-PCR data were analyzed by the 2^(−ΔΔCT)^ method using GAPDH as a reference gene.

### *In vivo* study model of colon cancer

All animal-related procedures were approved and carried out under the regulations and guidelines of the Oklahoma State University Animal Care and Use Committee. Female 10-week Balb/c mice (Charles River, Wilmington, MA) were inoculated with 0.5 × 10^6^ cells/50 μl in the thigh region using a 25-gauge needle (BD, Franklin Lakes, NJ, USA). Mice were monitored and tumor growth was measured by serial caliper measurements (General Tools Fraction+ ™, New York, NY, USA). Tumor volumes were calculated using the formula (length X width^2^)/2, where length is the largest dimension and width is the smallest dimension perpendicular to the length. When the tumors reached a volume of 400–500 mm^3^ mice, were randomized into 6 groups (n = 6–7/group). Treatment groups were designed as follows: Control+/− HIFU, *Salmonella*+/– HIFU, TB+/− HIFU. For *in vivo* treatment, 10^6^ *Salmonella* or TB were administered by intravenous injection, and HIFU was administered 24 h later. All mice were sacrificed 5 days after the injection and the tumor, liver and spleen and blood were collected for further processing.

### HIFU hyperthermia treatment set-up and methodology

For HIFU treatment, mice were anesthetized with 2–5% isoflurane and restrained in custom built mouse holders attached to a 3D positioning stage. An integrated ultrasound-HIFU Alpinion platform with 1.0 MHz central transducer frequency, 45 mm radius, and 64 mm aperture diameter with a central opening 40 mm in diameter was used for tumor identification and treatment. The mouse was oriented so that its dorsal side was facing the transducer and the caudal half was lowered into a 37 °C water bath. The path from the transducer to the tumor was aligned along the z-axis. The center of the tumor was aligned with the HIFU focus at a fixed focal depth for efficient coverage, and VIFU-2000 software was used to define the target boundary and slice distance in X, Y, and Z directions for automatic rastering of the transducer as demonstrated previously^21^. HIFU treatment parameters used were as follows: 35% duty cycle, 5 Hz PRF, and 6 W Power to achieve a mean target temperature of 40–42.5 °C at the focus. A 3 × 3 raster pattern was followed for hyperthermia treatment of tumors with HIFU. The distance between any two central focus points on a tumor was 2 mm to ensure that the entire volume was heated to ~42 °C. Each point (1 × 1 × 10 mm) within the raster pattern was heated 60 s. The total treatment duration was ~30 min. to cover the entire tumor.

### Post-treatment tissue analysis

Upon completion of treatment, mice were euthanized. Tumor and tissue samples from liver, spleen blood was collected for flow cytometry, qPCR and ELISA. Tumor and serum samples for qPCR and ELISA were snap-frozen over liquid nitrogen, and stored at −80 °C until analysis.

### Flow cytometric analysis

Tumor tissues were minced and digested in 200 U/ml collagenase IV buffer at 37 °C for 1.5 hours. The digested tissue was strained using a 70μm cell strainer (Corning Inc., Corning, NY) to obtain single cell suspensions. Cells were stained using antibody mixes, for different immune cell populations, prepared in 1X PBS with 2% FBS staining buffer, incubated for 1 hour at 4 degree C in dark. For IFNγ detection, staining buffer contained 0.1% saponin was used for permeabilization. The labeled cells were then fixed in 4% paraformaldehyde and analyzed using FACS Aria flow sorter (BD Biosciences, NJ) using the following panel - M1 macrophages (CD11b + F4/80 + CD86+), M2 macrophages (CD11b + F4/80 + CD206+), myeloid-derived suppressor cells (MDSCs)- granulocytic (CD11b + Ly6G + Ly6C−) and monocytic (CD11b + Ly6G- Ly6C+), and T cells (CD3 + CD4 + CD8 + IFNγ). UltraComp eBeads were used for compensation controls as per the manufacturer’s instructions. Fluorescence-minus-one (FMO) samples were used as negative controls. Data was analyzed using FlowJo software v.10.2 (Tree Star Inc, OR, USA). Data was expressed as cell number per g of tumor using the formula N = NS × NT/NA × W, where NS is cells of interest, NA - number of cells counted in flow cytometer (singlet tumor cells), and NT as tumor cells = 20000, W – weight of tumor.

### Determination of serum cytokine levels by ELISA

IL1β, TNFα and IL10 protein levels were measured in the serum by enzyme linked immunosorbent assay (R&D Inc., MN, USA – Quantikine ELISA), according to the manufacturer’s instructions. For serum separation, the whole blood was collected and allowed to clot at room temperature for 30 minutes. The clot was removed by centrifugation at 6000 × g for 20 minutes, at 4 °C. The serum was transferred into a new polypropylene tube and stored at −80 °C until used for ELISA cytokine analysis.

### Bacteria quantification

Aseptically collected tumors were individually placed in a pre-weighed sterile tube containing 500 μl of cold sterile PBS and placed on ice. Briefly, all tumor samples were homogenized and serially diluted with PBS and plated on LB agar plates. The colonies were enumerated and expressed as log CFU per gram of tumor.

### Statistical Analysis

The relative gene expression from real time quantitative PCR analysis was conducted with comparative C_T_ method (2^−ΔΔCT^) using GAPDH as a reference gene. Mean values for conditioned media treatment were compared to control with one way ANOVA and Tukey’s multiple comparisons post-hoc test.

Values were reported as a mean ± standard error of the mean (SEM) and the number of independent replicates is indicated in the figure legends. Treatment groups were compared for differences in mean using analysis of variance (ANOVA) followed by Tukey’s multiple comparisons posthoc test. All analyses were performed using GraphPad Prism 7.0 (GraphPad Software Inc.). All p-values were two-sided, and p < 0.05 was taken to indicate statistical significance.

### Data Availability Statement

The datasets generated during and/or analysed during the current study are available from the corresponding author on reasonable request.
